# 5-Lipoxygenase Inhibitors Attenuate TNF-α-Induced Inflammation in Human Synovial Fibroblasts

**DOI:** 10.1371/journal.pone.0107890

**Published:** 2014-09-17

**Authors:** Han-Ching Lin, Tzu-Hung Lin, Ming-Yueh Wu, Yung-Cheng Chiu, Chih-Hsin Tang, Mann-Jen Hour, Houng-Chi Liou, Huang-Ju Tu, Rong-Sen Yang, Wen-Mei Fu

**Affiliations:** 1 Department of Pharmacology, College of Medicine, National Taiwan University and Hospital, Taipei, Taiwan; 2 Department of Orthopaedics, Taichung Veterans General Hospital, Taichung, Taiwan; 3 Department of Pharmacology, College of Medicine, China Medical University, Taichung, Taiwan; 4 School of Pharmacy, College of Medicine, China Medical University, Taichung, Taiwan; 5 Department of Orthopedics, College of Medicine, National Taiwan University and Hospital, Taipei, Taiwan; Chang Gung University, Taiwan

## Abstract

The lipoxygenase isoform of 5-lipoxygenase (5-LOX) is reported to be overexpressed in human rheumatoid arthritis synovial tissue and involved in the progress of inflammatory arthritis. However, the detailed mechanism of how 5-lipoxygenase regulates the inflammatory response in arthritis synovial tissue is still unclear. The aim of this study was to investigate the involvement of lipoxygenase pathways in TNF-α-induced production of cytokines and chemokines. Human synovial fibroblasts from rheumatoid patients were used in this study. 5-LOX inhibitors and shRNA were used to examine the involvement of 5-LOX in TNF-α-induced cytokines and chemokines expression. The signaling pathways were examined by Western Blotting or immunofluorescence staining. The effect of 5-LOX inhibitor on TNF-α-induced chemokine expression and paw edema was also explored *in* *vivo* in C57BL/6 mice. Treatment with 5-LOX inhibitors significantly decreased TNF-α-induced pro-inflammatory mediators including interleukin-6 (IL-6) and monocyte chemo-attractant protein-1 (MCP-1) in human synovial fibroblasts. Knockdown of 5-LOX using shRNA exerted similar inhibitory effects. The abrogation of NF-κB activation was involved in the antagonizing effects of these inhibitors. Furthermore, 5-LOX inhibitor decreased TNF-α-induced up-regulation of serum MCP-1 level and paw edema in mouse model. Our results provide the evidence that the administration of 5-LOX inhibitors is able to ameliorate TNF-α-induced cytokine/chemokine release and paw edema, indicating that 5-LOX inhibitors may be developed for therapeutic treatment of inflammatory arthritis.

## Introduction

Rheumatoid arthritis (RA) is a chronic and systemic autoimmune syndrome, which is characterized by massive synovial proliferation and inflammation and leads to the destruction of joint cartilage and bone [1]. In addition, many kinds of cells infiltrate into the joint cavity during arthritis, including immune cells (such as macrophage, T cells and B cells) and erosive cells such as bone resorptive osteoclasts.

Arachidonic acid (AA) is a key inflammatory intermediate from the lipid composition. In response to a variety of stimuli, AA is released from membrane phospholipid by phospholipase. Pruzanski *et* *al*. [2] reported that the phospholipase A2 (PLA2) activity is increased in arthritis. Cytokines including TNF-α, IL-1 are reported to stimulate the activity of PLA2 [3], [4]. Both cyclooxygenase (COX) and lipoxygenase (LOX) pathways are involved in the inflammatory actions related to AA [5].

The inflammatory synovial fluid in RA patients contains many kinds of cytokines including high levels of leukotrienes [6]. Leukotriene B4 (LTB4) is a downstream product of 5-lipoxygenase (5-LOX) and LTB4 is reported to be produced mainly by neutrophils, macrophages and mast cells [7]. LTB4 is considered as a powerful proinflammatory chemotactic agent and has been implicated as an important mediator of joint inflammation in RA. There are higher levels of LTB4 in the serum of RA patients than patients with inactive arthritis or normal subjects [8]. Ahmadzadeh *et* *al*. [9] demonstrate that the serum levels of LTB4 correlate with the disease severity. In addition, neutrophil-derived LTB4 is reported to contribute to arthritis induction and severity in a mouse inflammatory arthritis model [10].

The 5-LOX cascades and the role of LTB4 in RA are well documented. 5-LOX is required for the production of leukotrienes (LTC4, LTD4, and LTE4) which are reported to be potent broncho-constrictors and proinflammatory mediators [11]. These leukotrienes are known as the cysteinly leukotrienes (cys-LTs). 5-LOX enzyme is also involved in the production of LTB4. This lipid is primarily synthesized in neutrophils and macrophages, where the enzyme LTA4 hydrolase converts LTA4 to LTB4 [12]. In addition, 5-LOX enzyme is also involved in the production of bioactive metabolites of 5-hydroxyeicosatetraenoic acid (5-HETE) and 5-oxo-6,8,11,14-eicosatetraenoic acid (5-oxoETE) [13], [14]. It has been reported that 5-LOX enzyme is present in the synovial lining of rheumatoid tissue [15]. Moreover, Gheroghe *et* *al*. [16] found that both 5-LOX and 15-LOX are present in RA and OA (osteoarthritis) synovium and 5-LOX is highly expressed in lining and sublining macrophages, neutrophils and mast cells.

PF-4191834, a novel selective 5-LOX inhibitor developed by Pfizer, is found to decrease arthritis-associated pain and inflammation in rat model [11]. However, the detailed mechanism of 5-LOX involved in inflammatory arthritis is still unclear. Here we found that blockade of 5-LOX by using commercial inhibitors or shRNA could decrease TNF-α-induced IL-6 and MCP-1 expression in human synovial fibroblasts. We then analyzed the signaling pathway and it was found that TNF-α-induced NF-κB activation was antagonized by 5-LOX inhibitors. In mouse model, it was also found that co-treatment of 5-LOX inhibitor could decrease TNF-α-induced MCP-1 serum level and paw edema. Our results demonstrate that 5-LOX is involved in TNF-α-induced inflammatory arthritis and may provide a new strategy for treating rheumatoid arthritis.

## Methods

### Ethics Statement

The study was approved by the Institutional Review Board of Taichung Veterans General Hospital (IRB TCVGH NO: C09248), and informed written consent was obtained from patients. All animal experiments were conducted in accordance with the Guidelines for Animals Research of Agriculture Council, ROC and approved by the Ethical Committee for Animal Research of the National Taiwan University (IACUC: 20120439).

### Materials

Mouse monoclonal antibody for α-tubulin, C23, NF-κB p65 and rabbit polyclonal antibody for IgG, IKKα/β, IκBα, NF-κB p50, NF-κB p65 and goat anti-mouse or anti-rabbit secondary antibody conjugated with horseradish peroxidase were purchased from Santa Cruz Biotechnology (Santa Cruz, CA, USA). Mouse monoclonal antibody for phosphor-IκBα and rabbit monoclonal antibody for phosphor-IKKα/β were from Cell Signaling Technology (Danvers, MA, USA). Rabbit anti-5-LOX antibody was from Novus (Littleton, CO, USA). 5-LOX inhibitors, including MK-886, Nordihydroguaiaretic acid (NDGA); leukotriene B4 and leukotriene B4 receptor antagonist LY29311 were from Cayman Chemical Company (Ann Arbor, MI, USA). Collagenase and 4′,6-diamidino-2-phenylindole (DAPI) were from Sigma-Aldrich (St. Louis, MO, USA). Recombinant human TNF-α and enhanced chemiluminescent HRP substrate (ECL) were from Millipore (Bedford, MA, USA). We purchased RPMI-1640 medium, trypsin and anti-rabbit secondary antibody conjugated with Alexa Fluor 488 from Invitrogen (Carlsbad, CA, USA) and fetal bovine serum (FBS) from Biological Industries (Kibbutz Beit Haemek, Israel). Tri-zol was from MDBIO (Taipei, Taiwan). MMLV Reverse Transcriptase kit was from Promega (Madison, WI, USA). Taqman PCR Master Mix and qPCR probes were from Applied Biosystems/Invitrogen (Foster city, CA, USA).

### Cell cultures

Human synovial fibroblasts were isolated by collagenase treatment from synovial tissues obtained from patients with rheumatoid arthritis (RA) undergoing total knee replacement surgeries (Taichung Veterans General Hospital, Taichung, Taiwan) [17]. Patients with RA were fulfilled with diagnostic criteria of American College of Rheumatology (ACR). Fresh synovial tissues were minced and digested in a solution of collagenase, and DNase. Isolated synovial fibroblasts were filtered through 70 µm nylon filters. The cells were then grown on culture dishes in 95% air-5% CO_2_ with RPMI-1640, which was supplemented with 10% heat-inactivated fetal calf serum, 100 U/ml penicillin, 100 µg/ml streptomycin, and 250 ng/ml fungizone (pH 7.6). Over 90% cultured cells were fibroblasts which were characterized by flow cytometry by using CD90 (Thy-1) antibody [18]. Synovial fibroblasts of passages four to nine were used in this study.

### Quantitative real-time PCR

Total RNA were extracted from human synovial fibroblasts using a Tri-zol kit. The absorbance was measured in a spectrophotometer, Picodrop (Picodrop Ltd., Essex, UK) at 260 and 280 nm. RNA was used for RT-PCR by using two-step MMLV RT kit. Gene expression was detected by Real-Time PCR which was executed by using a SYBR Green PCR Master Mix (Applied Biosystems, Foster city, CA, USA) and an ABI StepOnePlus Real-time PCR system (Applied Biosystems). The cDNA was amplified with gene specific primers as shown below:

GAPDH: [19].

Forward primer: TGGGTGTGAACCATGAGAAG.

Reverse primer: GCTAAGCAGTTGGTGGTGC.

IL-6: [20].

Forward primer: TCACTGGTCTTTTGGAGTTTGA.

Reverse primer: AGAGCCCTCAGGCTGGACT.

MCP-1/CCL-2: [21].

Forward primer: ATGCAATCAATGCCCCAGTC.

Reverse primer: TGCAGATTCTTGGGTTGTGG.

Amplification was performed in the following cycling conditions: 50°C for 2 min and 95°C for 10 min and then 40 cycles at 95°C for 15 s followed by 60°C for 1 min. The optimal concentrations of primers and templates used in each reaction were established based on the standard curve created before reaction and corresponding to nearly 100% efficiency of the reaction. The reference household gene used to normalize the amount of mRNA was GAPDH. The fold change in gene expression relative to control was calculated by 2^−ΔΔCT^.

### Measurement of cytokines and chemokines

After treatment with TNF-α or test substances, the levels of IL-6 or MCP-1 in the culture medium of human synovial fibroblasts and serum from mice were determined by enzyme-linked immunosorbent assay (ELISA). The conditioned medium or serum from mice was obtained and IL-6 or MCP-1 was detected according to the manufacturer’s protocol (R&D Systems, Minneapolis, MN, USA). All independent experiments were performed and the absorbance was determined using microplate reader (Bio-Tek, Winooski, VT, USA).

### Transfection of RNA interference

The 5-LOX-shRNA conjugated on the vector of pLKO.1 with ampicillin-resistant region was purchased from National RNAi Core Facility (RNAi Core, Taipei, Taiwan). 3 µg 5-LOX-shRNA and 6 µl Lipofectamine 2000 (Invitrogen, Carlsbad, CA, USA) were premixed with OPTI-MEM separately for 5 min and then mixed with each other for 25 min and then applied to human synovial fibroblasts. The empty vector of shRNA was used as negative control. After 36 hr, the cells were harvested or the culture medium was replaced with serum-free medium and test substances.

### Western Blot

After treatment with test substances, synovial fibroblasts were then washed with cold PBS and lysed for 30 min at 4°C with lysis buffer as described previously [18]. For the separation of cytoplasmic extracts (CE) and nuclear extracts, cells were cultured onto 10 cm dish. After reaching confluence, cells were treated with test substances, cytoplasmic extracts and nuclear extracts were separated by NE-PER (Thermo Scientific-Pierce, Rockford, IL, USA). Equal protein (30 µg) was applied per lane, and electrophoresis was performed under denaturing conditions on a 8% or 12% SDS gel and transferred to nitrocellulose membranes (Invitrogen). The blots were blocked with 5% non-fat milk in TBS-T (0.5% Tween 20 in 20 mM Tris and 137 mM NaCl) for 1 h at room temperature and then probed with antibodies against 5-LOX, phosphor-IκBα, IκBα, phospho-IKKα/β, IKKα/β, NF-κB p50, NF-κB p65 (1∶1000) at 4°C overnight. After 3 washes by TBS-T, the blots were subsequently incubated with goat anti-rabbit or anti-mouse peroxidase-conjugated secondary antibody (1∶10000) for 1 h at room temperature. The blots were visualized by enhanced chemiluminescence using Amersham HyperfilmTM ECL (GE Healthcare, Pollards Wood, UK) or Biospectrum Imaging System (UVP, Upland, CA, USA). For normalization purposes, the same blot was also probed with mouse anti-α-tubulin antibody or mouse anti-C23 antibody (1∶1000).

### Immunofluorescent staining

For immunolabeling studies, synovial fibroblasts were seeded on glass overnight and then treated with test substances. After TNF-α (25 ng/ml) treatment for 30 min with or without inhibitors, 4% paraformaldehyde was used to fix the cells for 15 min. 0.1% triton was used to permeabilize the cells and 4% BSA was used for blocking non-specific binding for 1 hr. Synovial fibroblasts were stained with primary mouse monoclonal antibody against NFκB-p65 (1∶200) (Santa Cruz sc-8008) (Lin *et* *al*., 2011) overnight and then with Alexa Fluor 488-conjugated goat anti-mouse secondary antibody (Invitrogen, Carlsbad, California). The nucleus was counterstained by DAPI and the confocal images were obtained by using excitation wavelength 494 nm and emission wavelength 519 nm, respectively (for Alexa-488) (model SP5 TCS; Leica, Heidelberg, Germany) and the thickness of optical sections was set at 0.8 µm.

### TNF-α-induced inflammation in animal models

8 weeks-old male C57BL/6 mice and 15-LOX knockout mice were purchased from the Laboratory Animal Center of National Taiwan University. All mice were kept under standard temperature, humidity, and timed lighting conditions and provided mouse chow and water ad libitum.

Recombinant mouse TNF-α (40 µg/kg) in the presence or absence of 5-LOX inhibitor NDGA (10 mg/kg) was injected intravenously from femoral vein of male C57BL/6 mice. Mice were sacrificed 6 hr later and serum was collected to perform ELISA analysis.

For the paw swelling experiment, TNF-α (200 ng dissolved in 10 µl PBS) in the presence or absence of NDGA (10 µM) or MK-886 (10 µM) was injected intraplantarly into the right hind paw and the PBS-injected left hind paw was used as control. Paw thickness was measured with digimatic caliper (Mitutoyo, Japan). The change of paw thickness was compared to the paw thickness at 0 hr.

### Statistics

The data given were means ± S.E.M. The significance of difference between the experimental group and control was assessed by one-way analysis of variance (ANOVA) and 2-tailed Student’s t-test. The difference is significant if the *p* value is less than 0.05.

## Results

### Effect of 5-LOX inhibitors on TNF-α-induced IL-6 expression in human synovial fibroblasts

**Figure 1:**
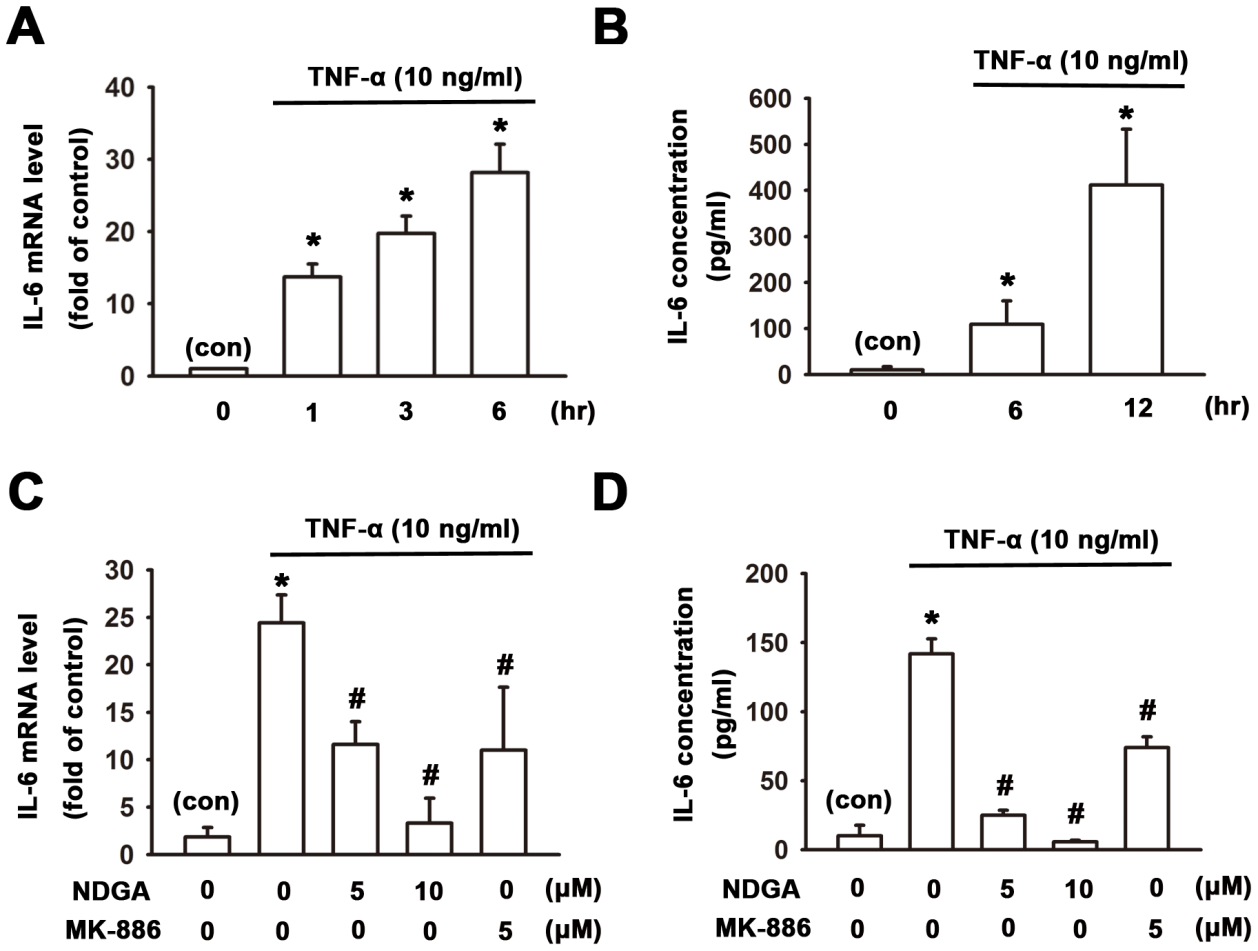
Inhibition of TNF-α-induced IL-6 expression by 5-LOX inhibitors in human synovial fibroblasts. Human synovial fibroblasts were incubated with TNF-α (10 ng/ml) for indicated time intervals. mRNA and released IL-6 were determined by QPCR and ELISA respectively. Treatment with TNF-α enhanced the IL-6 mRNA (A) expression and cytokine release (B) in a time-dependent manner. Pretreatment with two 5-LOX inhibitors, NDGA (5 or 10 µM) or MK-886 (5 µM) for 1 hr significantly inhibited the TNF-α-induced mRNA (C) or protein levels (D) of IL-6. Data are presented as mean ± SEM. *, p<0.05 compared with vehicle control (con). #, p<0.05 compared with TNF-α treatment alone.

In inflammatory arthritis, synovial fibroblasts contribute to synovium inflammation, angiogenesis, matrix degradation by its abilities to produce inflammatory cytokines, matrix degradation enzymes and angiogenic factor [22]. In addition, TNF-α is the potent inflammatory cytokine and is responsible for synovial fibroblasts-involved cartilage degradation. First of all, we examined whether TNF-α can enhance the production of pro-inflammatory cytokine, IL-6. Human synovial fibroblasts were incubated with TNF-α (10 ng/ml) in serum-free medium for several time intervals. The mRNA and released protein level of IL-6 were evaluated by quantitative PCR and ELISA, respectively. Figures 1A and 1B showed that treatment with TNF-α enhanced the mRNA and protein levels of IL-6 in a time-dependent manner. Treatment of TNF-α for 6 hr increased mRNA expression of IL-6 to 28.2±3.9 fold of control (Figure 1A). In addition, treatment of TNF-α also increased IL-6 protein release to 9.9±2.3 fold and 40.0±7.0 fold of control at 6 or 12 hr, respectively (Figure 1B).

To investigate the role of 5-LOX in rheumatoid arthritis, human synovial fibroblasts were pretreated with 5-LOX inhibitors NDGA (5 and 10 µM) and MK-886 (5 µM) for 1 hr and then treated with TNF-α (10 ng/ml) for next 6 hr. mRNA extracted from cell lysates was evaluated by quantitative PCR and the conditioned medium collected was measured for IL-6 protein release by ELISA assay. Real-time PCR analysis showed that TNF-α significantly increased IL-6 mRNA levels and pretreatment with 5-LOX inhibitors NDGA (5 and 10 µM) and MK-886 (5 µM) could inhibit the upregulatory effect of TNF-α by 54.7±6.0%, 91.4±4.3% and 57.3±12.6%, respectively (Figure 1C). In addition, treatment with TNF-α increased the secreted IL-6 in the conditioned medium and pretreatment of 5-LOX inhibitors NDGA (5 and 10 µM) and MK-886 (5 µM) decreased the TNF-α-induced IL-6 protein levels by 83.0±1.1%, 96.3±0.4% and 48.1±2.8%, respectively (Figure 1D).

### Effect of 5-LOX inhibitors on TNF-α-induced MCP-1/CCL-2 expression in human synovial fibroblasts

**Figure 2:**
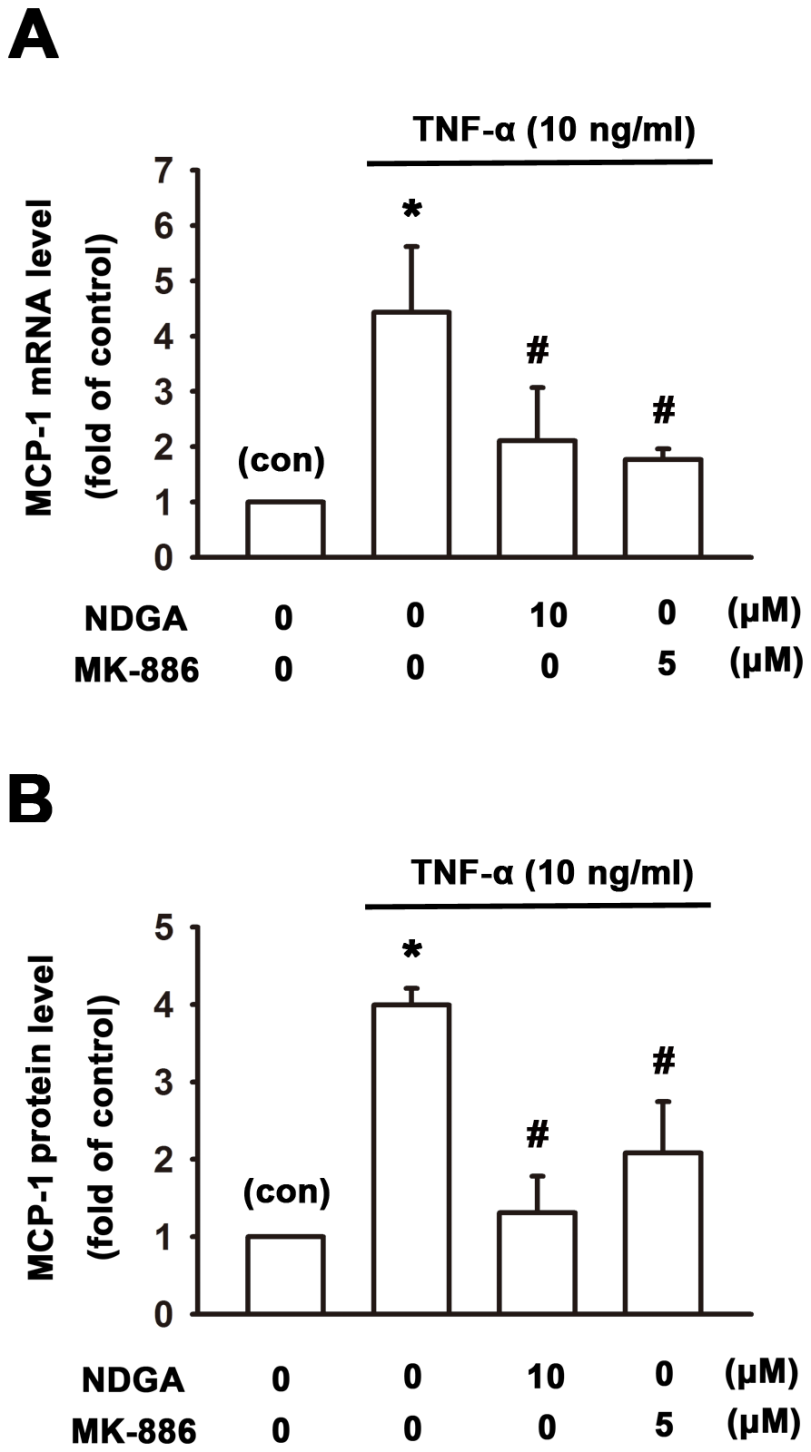
Attenuation of TNFα-induced MCP-1/CCL-2 expression by 5-LOX inhibitors in human synovial fibroblasts. Human synovial fibroblasts were pretreated with NDGA (10 µM) or MK-886 (5 µM) for 1 hr, and then treated with TNF-α for another 6 hr. Cell lysate was extracted for real-time PCR evaluation (A) and conditioned medium was collected for ELISA measurement of MCP-1 (B). Note that 5-LOX inhibitors NDGA and MK-886 antagonized TNF-α-induced mRNA and protein levels of MCP-1/CCL-2. Data are presented as mean ± SEM. *, p<0.05 compared with control (con). #, p<0.05 compared with TNF-α-treatment alone.

One of potential therapy in RA is targeting chemokines and a randomized clinical trial with an anti-CCL2/MCP-1 monoclonal antibody in RA patients has been reported previously [23]. MCP-1 plays a key role in leukocyte migration and recruitment of mononuclear phagocytes during inflammation in the joint and previous study indicated that there is overproduction of CCL-2/MCP-1 in RA patient’s synovium [24]. To further elucidate the anti-inflammatory effects of 5-LOX inhibitors in the rheumatoid arthritis, human synovial fibroblasts were pretreated with 5-LOX inhibitors NDGA (10 µM) and MK-886 (5 µM) for 1 hr and then treated with TNF-α (10 ng/ml) for another 6 hr. mRNA levels extracted from cell lysates was evaluated by Real-time PCR and the supernatant was collected for measuring MCP-1 protein levels by ELISA assay. Real-time PCR analysis showed that TNF-α significantly increased MCP-1 mRNA expression to 4.5±0.5 fold of vehicle control (Figure 2A). Pretreatment of 5-LOX inhibitors NDGA (10 µM) and MK-886 (5 µM) decreased TNF-α-induced MCP-1 mRNA levels by 70.6±11.0%, and 77.9±3.2%, respectively (Figure 2A). The released MCP-1 was analysed by ELISA analysis and showed that treatment with TNF-α increased the secreted MCP-1 in the conditioned medium to 4.0±0.1 fold of vehicle control (Figure 2B). Pretreatment of 5-LOX inhibitors NDGA (10 µM) and MK-886 (5 µM) antagonized TNF-α-induced MCP-1 protein levels by 89.6±9.1%, and 63.8±12.7%, respectively (Figure 2B).

### Involvement of leukotriene B4 in TNF-α-induced cytokine expression in human synovial fibroblasts

**Figure 3:**
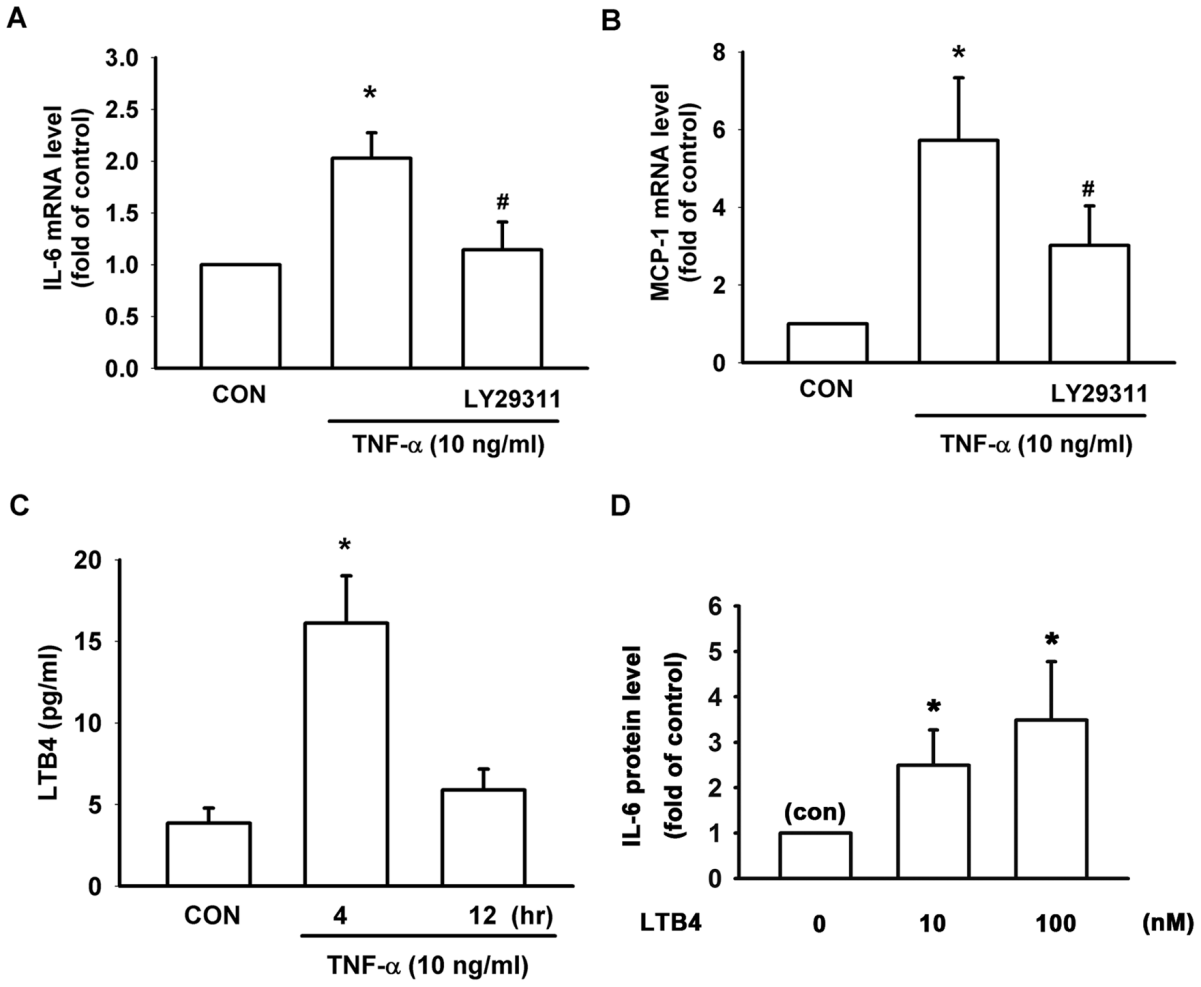
Involvement of leukotriene B4 in TNF-α-induced cytokine expression in human synovial fibroblasts. Human synovial fibroblasts were pretreated with LTB4 receptor antagonist LY29311 (1 µM) for 1 hr, and then treated with TNF-α for another 6 hr. TNF-α-induced up-regulation of IL-6 and MCP-1 were antagonized by LY29311 (A, B). RASFs were treated with TNF-α (10 ng/ml) for 4 and 12 hr, and the conditioned medium was collected for ELISA measurement of LTB4. Data show that LTB4 was increased at 4 hr (C). (D) Human synovial fibroblasts were treated with leukotriene B4 for 6 hrs at different concentrations, and conditioned medium was collected for ELISA assay. ELISA analysis showed that treatment with leukotriene B4 enhanced IL-6 release in a concentration-dependent manner. Data are presented as mean ± SEM. *, p<0.05 compared with control (con). #, p<0.05 compared with TNF-α-treatment alone.

Leukotriene B4 (LTB4) is one of 5-Lipoxygenase downstream metabolites and plays an important role in pathogenesis of RA [25]. Recent study has suggested that LTB4 is an essential and non-redundant role in both acute and chronic inflammation of rheumatoid arthritis [10]. In addition, exogenous LTB4 could increase the expression of cytokines like TNF-α and IL-1β in human synovial fibroblasts [26]. We thus investigated whether LTB4 is involved incytokines release in human synovial fibroblasts. Human synovial fibroblasts were pretreated with LTB4 receptor antagonist LY29311 (1 µM) for 1 hr and then treated with TNF-α (10 ng/ml) for another 6 hr. mRNA were extracted for Real-time PCR analysis, and the results show that TNF-α-induced up-regulation of IL-6 and MCP-1 were reduced by LY29311 (Figure 3A, 3B). In addition, treatment of TNF-α for 4 hr increased the secretion of LTB4 from 3.9±0.9 to 16.1±2.9 (pg/ml) (Figure 3C). Treatmentwith LTB4 at different concentrations in serum-free RPMI medium for 6 hr and the quantity of IL-6 in conditioned medium was measured. It was found that treatment with LTB4 (10, 100 nM) increased secreted IL-6 protein levels to 2.5±0.5-fold and 3.5±0.3 fold of control, respectively (Figure 3D).

### Knockdown of 5-LOX inhibits TNF-α-induced IL-6 and MCP-1 release in human synovial fibroblasts

**Figure 4:**
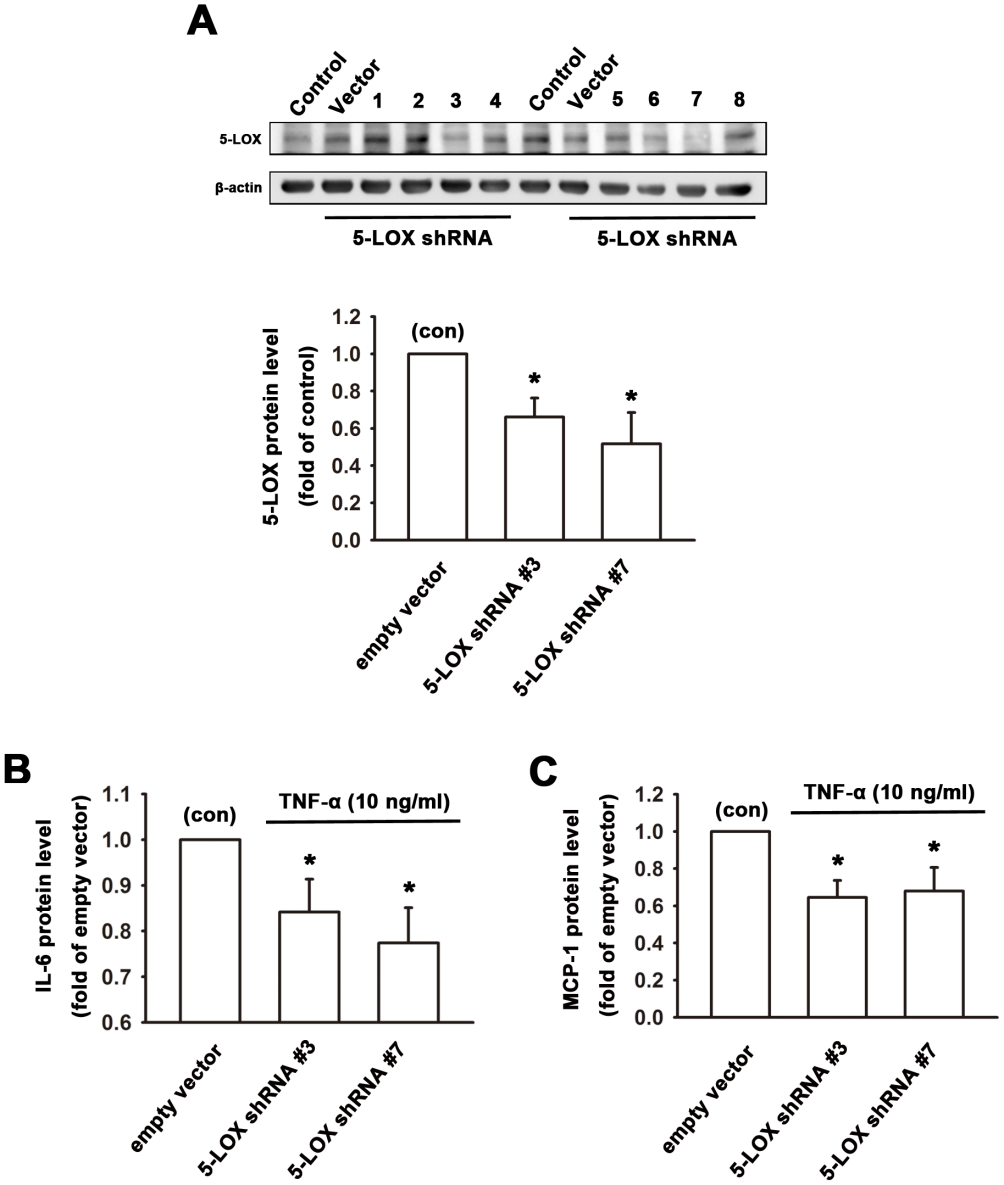
Knockdown of 5-LOX inhibits TNF-α-induced IL-6 and MCP-1 release in human synovial fibroblasts. Total protein extracts from RASFs transfected with empty vector or 5-LOX shRNA were used for Western blotting. (A) Immunoblotting showed that cells transfected with 5-LOX shRNA of clone No.3 and No.7 markedly decreased 5-LOX expression. Human synovial fibroblasts transfected with empty vector or 5-LOX shRNA were treated with TNF-α for 6 hr. IL-6 and MCP-1 protein release in the conditioned medium was evaluated by ELISA. Note that knockdown of 5-LOX decreased IL-6 (B) and MCP-1 (C) release following TNF-α treatment for 6 hr. Data are presented as mean ± SEM. *, p<0.05 compared with empty vector control (con).

To further examine the role of 5-LOX in TNF-α-induced IL-6 and MCP-1 expression, 5-LOX knockdown was performed by using 5-LOX shRNA and the protein release in the conditioned medium in response to TNF-α administration was measured. Total protein extracts from RASFs transfected with empty vector or 5-LOX shRNA were analyzed by immunoblotting. Figure 4A showed that cells transfected with 5-LOX shRNA No.3 and No.7 significantly decreased 5-LOX expression. Human synovial fibroblasts transfected with empty vector or 5-LOX shRNA clone No.3 and No.7 were then used to treat with TNF-α for 6 hr. IL-6 and MCP-1 protein release in the conditioned medium was evaluated by ELISA analysis. It was found that knockdown of 5-LOX with shRNA No.3 and No.7 decreased TNF-α-induced IL-6 protein release by 16.8±4.7% and 23.8±4.9%, respectively (Figure 4B). In addition, knockdown of 5-LOX with shRNA No.3 and No.7 reduced TNF-α-induced MCP-1 protein release by 36.6±5.2% and 32.6±7.9% following 6 hr treatment with TNF-α (Figure 4C).

### Effect of 5-LOX inhibitors on TNF-α-induced IKKα/β activation, IκBα phosphorylation, IκBα degradation in human synovial fibroblasts

**Figure 5:**
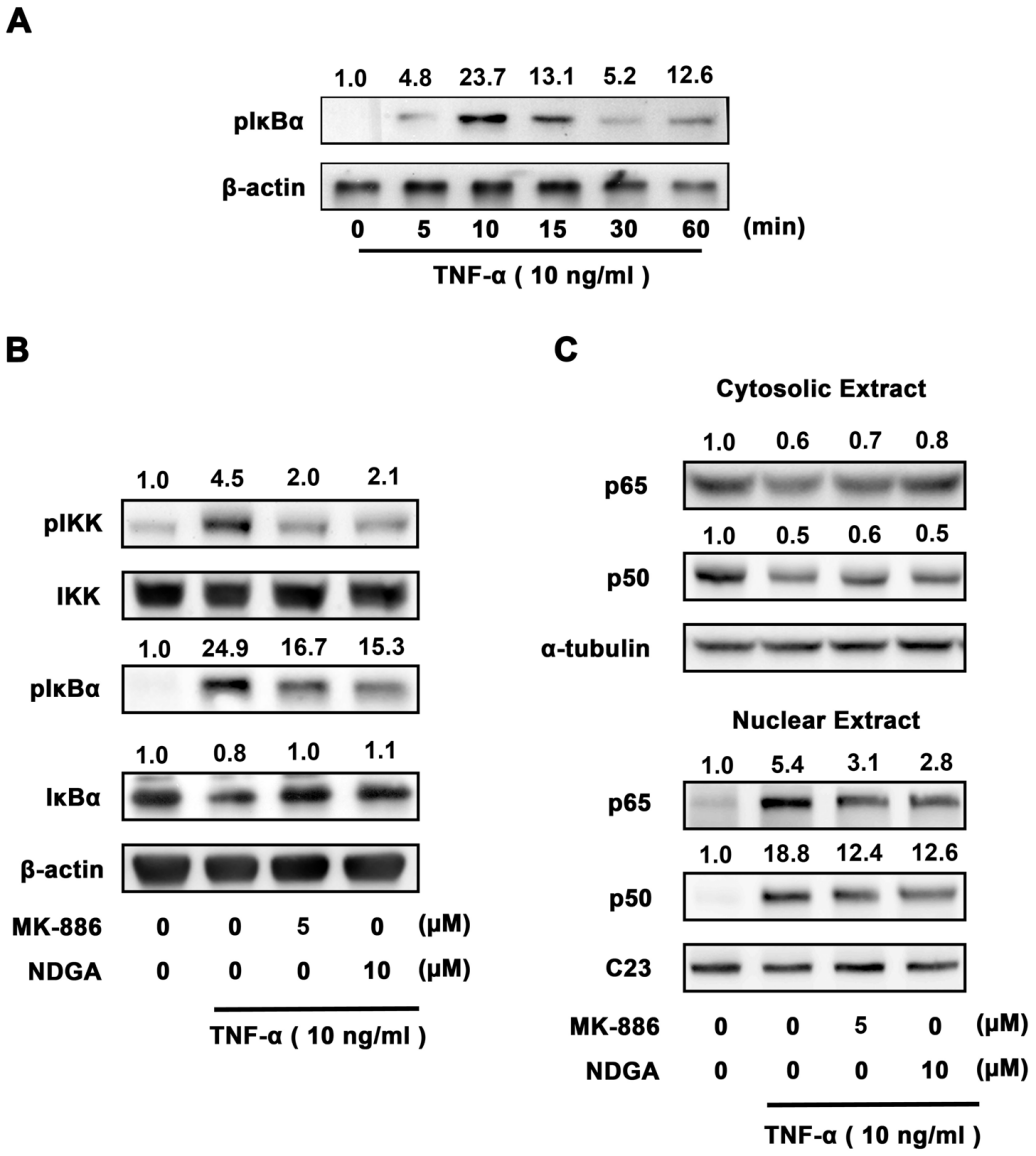
5-LOX inhibitors antagonize TNF-α-induced IKKα/β activation, IκBα phosphorylation, IκBα degradation and NF-κB nuclear translocation in human synovial fibroblasts. (A) Immunoblotting showed that treatment of TNF-α (10 ng/ml) enhanced the phosphorylation of IκBα time-dependently in human synovial fibroblasts. (B) Human synovial fibroblasts were pre-incubated with 5-LOX inhibitors NDGA (10 µM) or MK-886 (5 µM) for 1 hr and then exposed to TNF-α for another 10 min. Note that pretreatment with NDGA or MK-886 could antagonize TNF-α-induced effects. (C) Human synovial fibroblasts were pretreated with NDGA (10 µM) or MK-886 (5 µM) for 1 hr. TNF-α (10 ng/ml) was then added for another 30 min. Cytosolic and nuclear extracts were separated by NE-PER kit. Note that NDGA or MK-886 significantly antagonized the nuclear translocation of NF-κB subunits of p65 and p50. C23 was used as nucleus marker.

**Figure 6:**
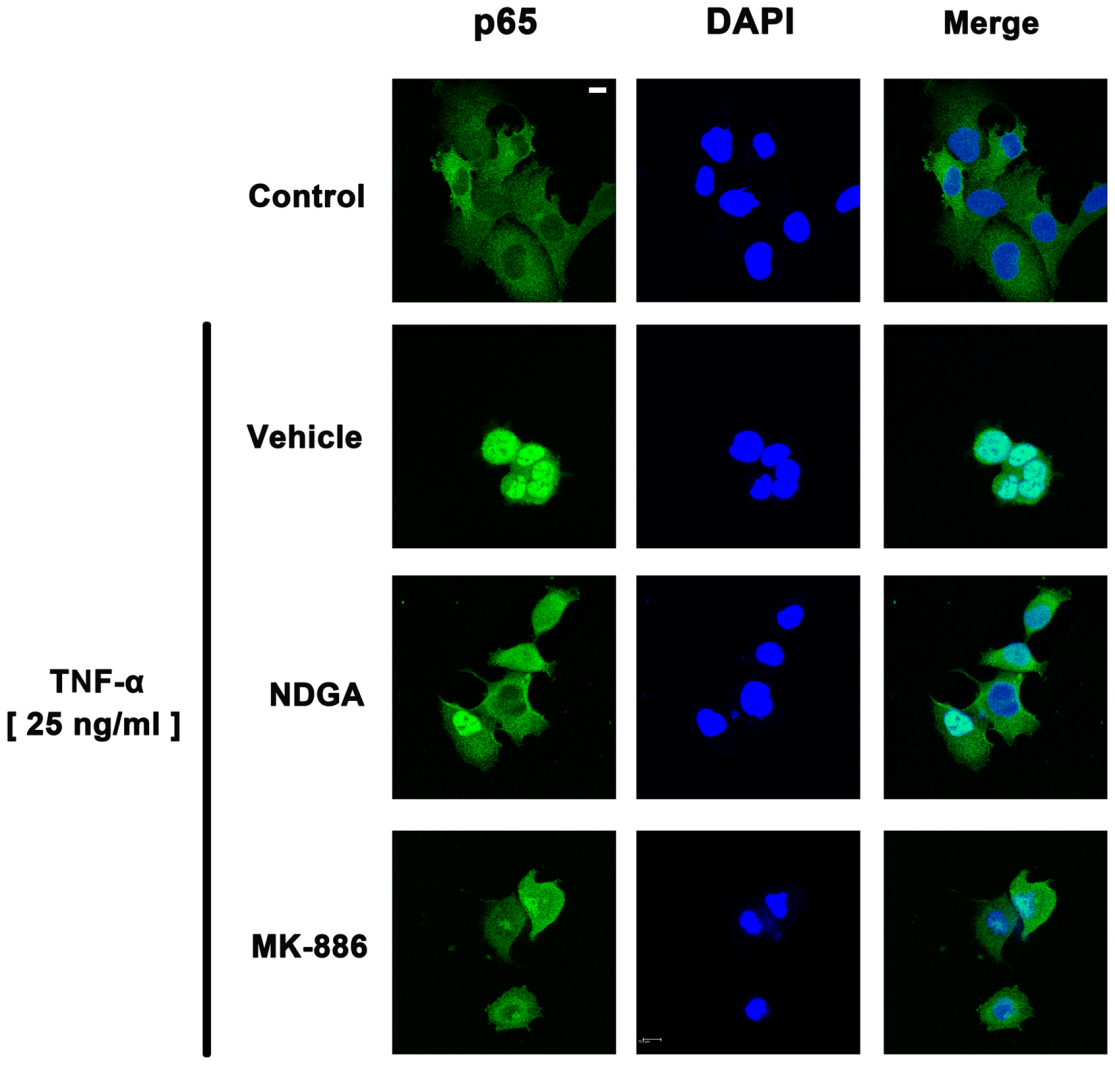
5-LOX inhibitors antagonize TNF-α-induced nuclear translocation of p65. Immunofluorescent staining showed that p65 translocated into nucleus after treatment of TNF-α (25 ng/ml) for 30 min. Pretreatment of 5-LOX inhibitors NDGA (10 µM) or MK-886 (5 µM) antagonized the nuclear translocation of p65. DAPI staining was used to indicate the location of nucleus. Scale: 5 µm.

NF-κB is implicated in the transcriptional regulation of inflammatory response by TNF-α. Bondeson *et* *al*. [27] have demonstrated that the activation of NF-κB plays an important role in the inflammatory action of TNF-α in synovial fibroblasts. To examine whether NF-κB pathway is involved in the antagonism of 5-LOX inhibitors on TNF-α-induced cytokine expression in human synovial fibroblasts, IKKα/β activation, IκBα phosphorylation, and IκBα degradation were evaluated by immunoblotting after stimulation by TNF-α. Since the canonical NF-κB pathway is induced by TNF-α and this activation leads to the recruitment and activation of an IKK complex comprising IKK alpha and/or IKK beta catalytic subunits. The IKK complex then phosphorylated IκBα at ser32 and ser36 to produce ubiquitination of IκBα at lysine residues and subsequent degradation by the 26 s proteasome. NF-κB complex (p50 and p65), which is associated with IκB to retain in the cytosol under resting state, is released and translocates to the nucleus [28]. Therefore, we first investigated the time-course of IκBα phosphorylation in human synovial fibroblasts. Cells were treated by TNF-α (10 ng/ml) for 5, 10, 15, 30, and 60 min and total proteins were extracted for Western blotting. The results showed that treatment with TNF-α (10 ng/ml) enhanced the phosphorylation of IκBα time-dependently in human synovial fibroblasts, and the phosphorylation of IκBα reached a peak at 10 min (Figure 5A). Human synovial fibroblasts were then pre-incubated with 5-LOX inhibitors NDGA (10 µM) or MK-886 (5 µM) for 1 hr and then exposed to TNF-α for another 10 min. It was found that pretreatment with NDGA or MK-886 could antagonize TNF-α-induced IKKα/β activation, IκBα phosphorylation and IκBα degradation (Figure 5B). Pretreatment of NDGA or MK-886 also inhibited the TNF-α-induced nuclear translocation of NF-κB subunits of p50 and p65 (Figure 5C). Immunofluorescence staining was also used to evaluate whether 5-LOX antagonized TNF-α-induced NF-κB nuclear translocation. Human synovial fibroblasts were treated with TNF-α (25 ng/ml) for 30 min and p65 translocated into nucleus (Figure 6). Pretreatment with 5-LOX inhibitors, including NDGA (10 µM) and MK-886 (5 µM) for 1 hr antagonized TNF-α-induced nuclear translocation of p65 (Figure 6). These results indicated that 5-LOX inhibitors decreased TNF-α-induced cytokine/chemokine expression via the abrogation of NF-κB signalling.

### Effect of 5-LOX inhibitors on TNF-α-induced monocyte chemo-attractant protein-1 (MCP-1) release and paw edema in animal model

**Figure 7:**
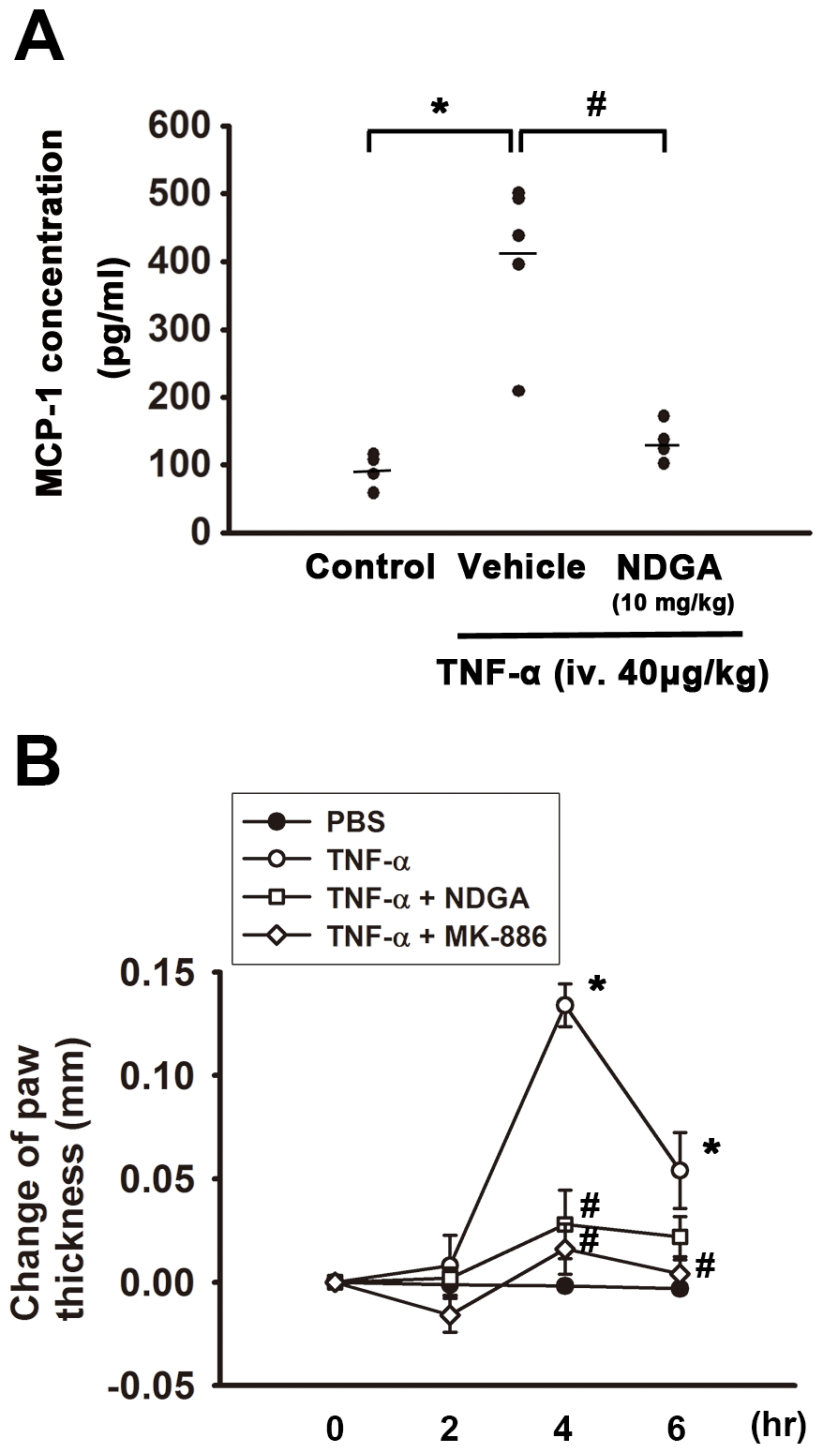
Antagonism by 5-LOX inhibitors on TNF-α-induced up-regulation of serum monocyte chemo-attractant protein-1 (MCP-1) level and paw edema in mice. (A) Male C57BL/6 mice (8 weeks-old) were injected with TNF-α (40 µg/kg in 0.2 ml saline) into femoral vein and 5-LOX inhibitor NDGA (10 mg/kg) was co-treated in another cohort. After 6 hr, mice were sacrificed and serum was collected for ELISA analysis. Note that mice injected with TNF-α markedly increased MCP-1 levels in serum, which was inhibited by co-treatment with 5-LOX inhibitor NDGA (10 mg/kg) (n = 4–5). (B) TNF-α was intraplantarly injected onto the paw of male C57BL/6 mice (8 weeks-old). 5-LOX inhibitors, NDGA (10 µM) or MK-886 (10 µM) was co-injected with TNF-α (10 µl). It was found that TNF-α induced acute paw edema and increased paw thickness during 4–6 hr. Note that co-administration of NDGA or MK-886 significantly attenuated TNF-α-induced paw edema (n = 5). Data are presented as mean ± SEM. *, p<0.05 compared with vehicle control. #, p<0.05 compared with TNF-α-treatment alone.

We have elucidated the role of 5-LOX in TNF-α-induced cytokines and chemokines production *in* *vitro*. We then examined the effect of 5-LOX inhibitor in animal model. Male C57BL/6 mice were injected with TNF-α (40 µg/kg, via femoral vein) with or without 5-LOX inhibitor NDGA (10 mg/kg). After 6 hr, mice were sacrificed and serum was collected to perform ELISA analysis. It was found that mice injected with TNF-α significantly increased MCP-1 levels in serum to 4.4±0.6 fold of vehicle-treated mice. However, co-treatment TNF-α with 5-LOX inhibitor NDGA (10 mg/kg) significantly reduced TNF-α-induced MCP-1 protein release in serum by 67.9±4.3% (Figure 7A). Moreover, we examined the effects of 5-LOX inhibitors on TNF-α-induced acute paw edema in mice model. As shown in figure 7B, intraplantar injection of TNF-α-increased the paw thickness and the thickness reached the peak at 4 hr. Co-administration with 5-LOX inhibitors, NDGA (10 µM) or MK-886 (10 µM) could markedly ameliorate TNF-α-induced paw edema. These results suggest that 5-LOX inhibitor can reduce TNF-α-induced chemokine production and paw edema *in* *vivo*.

## Discussion

Synovial fibroblasts play a pivotal role in early events of rheumatoid arthritis [29]. Many factors contribute to synovial fibroblasts activation and enhance the bone destruction [22]. Various reports have indicated that cytokines such as TNF-α, IL-1β and IL-6 play key roles in driving the inflammation and synovial cell proliferation characterizing rheumatoid arthritis-associated joint destruction [30]. Moreover, synovial fibroblasts have capacities to produce and secrete a wide range of pro-inflammatory mediators, including cytokines like IL-1β, IL-6, TNF-α and chemokines like MCP-1, MIP-1a [31]. Inflammatory cytokines also can induce the release of additional inflammatory factors and enhance the activation of synovial fibroblasts [32]. Furthermore, cytokines themselves can further promote more inflammatory cytokines and chemokines in synovial fibroblasts, amplifying inflammatory events in the synovium [33]. Therefore, it is important to ameliorate the activation of synovial fibroblasts in the treatment of inflammatory arthritis.

Current therapies of biologic agents for rheumatoid arthritis have targeted these cytokines. Despite of the good efficacy of biologic agents such as TNF-α or IL-1β blocker for many patients with rheumatoid arthritis, augmented risk of some adverse side effects such as infection [34] and malignancy [35] still persist in concern. In addition, these biological therapies such as etanercept, abatacept, rituximab, and tocilizumab for rheumatoid arthritis directly target immune cells such as T and B cells and pro-inflammatory cytokines. It indicates that other therapies and new targets in the treatment of RA, especially ones targeting non-immune cells like synovial fibroblasts may be possible.

5-lipoxygenase is reported to be present in RA synovium and mostly expressed in macrophages, neutrophils and mast cells in the sublining layer [16]. Here we found that synovial fibroblasts isolated from rheumatoid arthritis patients express high protein levels of 5-LOX which is consistent with high levels of leukotriene B4, a 5-LOX downstream metabolite, found in synovial fluid in rheumatoid arthritis patients [6]. Leukotrienes are regarded as inflammatory mediators derived from the 5-LOX cascade of arachidonic acid [36] and implicated in the pathogenesis of several human acute and chronic inflammatory diseases such as atherosclerosis, dermatitis, cancer and rheumatoid arthritis [36]. Unlike diseases such as asthma, allergic rhinitis, and ischemia/stroke that associated with an overproduction of CysLTs, rheumatoid arthritis is more linked to an overproduction of LTB4, another branch downstream metabolite of 5-LOX [37]. LTB4 has long been considered to have deleterious effects in arthritis. In atherosclerosis, LTB4 can induce the overexpression of TNF-α, IL-6 and MCP-1 mRNA in cultured monocytes, causing an inflammatory environment [38]. In addition, we also found that exogenous LTB4 increased IL-6 protein release in a concentration-dependent manner in synovial fibroblasts, and the TNF-α-induced up-regulation of IL-6 and MCP-1 were antagonized by the pretreatment of leukotriene B4 receptor antagonist LY29311. TNF-α is reported to be involved in the arachidonic acid metabolism. TNF-α at 1 nM stimulates the lipoxygenase pathway, and 5-LOX metabolites including LTB4 and 5-HETE increase in human osteoblastic osteosarcoma cells [39]. Moreover, in immune inflammation, it is found that TNF-α is involved in ovalbumin-induced neutrophil migration through a LTB4-dependent mechanism so that MK-886, a 5-LOX inhibitor, inhibits the TNF-α-induced neutrophil migration. TNF-α can also stimulate the secretion of LTB4 from peritoneal cells [40]. LTB4 also promotes TNF-α-induced CCL27 expression via the NF-κB pathway in human keratinocytes [41]. Here we found that 5-LOX inhibitors, including MK-886 and NDGA significantly antagonized TNF-α-induced IL-6 and MCP-1 mRNA expression and protein release in a concentration-dependent manner in human synovial fibroblasts. In addition, it was also found that knockdown of 5-LOX by shRNA transient transfection reduced the protein release of IL-6 and MCP-1 in synovial fibroblasts, indicating that 5-LOX pathway or even its downstream metabolites play a crucial role in TNF-α-induced cytokine and chemokine upregulation in human synovial fibroblasts. These findings are also consistent with previous studies that mice lacking 5-LOX or treatment with 5-LOX inhibitor are protected from inflammatory arthritis [10].

NF-κB is activated during the early stage of joint inflammation in human synovial tissue [42]. LTB4, the downstream metabolite of 5-LOX, increases the mRNA expression of IL-6 and MCP-1 via BLT1 and BLT2, G protein-coupled receptors of leukotrienes, via a NF-κB-dependent mechanism in atherosclerosis [38]. Moreover, LTB4 may amplify the NF-κB activation through Stat1-dependent expression of MyD88 and reducing SOCS1 inhibition of MyD88 in mouse macrophages [43]. 5-LOX also plays a key role in LPS-induced monocyte adhesion to vascular endothelium by increasing expression of Mac-1 via NF-κB signaling pathways [44], indicating that 5-LOX enzyme activities or its downstream metabolites may be linked to NF-κB pathway. Here we found that 5-LOX inhibitors including MK-886 and NDGA antagonized TNF-α-induced IKKα/β activation, IκBα phosphorylation and IκBα degradation in human synovial fibroblasts. Furthermore, 5-LOX inhibitors also antagonized TNF-α-induced nuclear translocation of NF-κB subunits of p65 and p50 in Western blotting and immunofluorescent staining. Moreover, IκBα phosphorylation and TNF-α-induced nuclear translocation of NF-κB subunits of p65 were also antagonized by leukotriene B4 receptor antagonist LY29311 (figure S1). These results suggest that 5-LOX and its downstream products are involved in TNF-α-induced cytokines and chemokines expression via the inhibition of NF-κB signaling pathways in human synovial fibroblasts.

Here we found that mice treated with NDGA significantly reduced TNF-α-induced up-regulation of MCP-1 in the serum and TNF-α-induced paw edema, whereas 5-LOX inhibitor could not completely reverse TNF-α-induced expression of IL-6 and MCP-1, indicating that other signals are involved in this action. In addition to LOX, arachidonic acid could also be metabolized by COX-2 to produce the bioactive eicosanoids, which play another pivotal part in the inflammatory arthritis. Developing novel 5-LOX inhibitor or 5-LOX/COX-2 dual inhibitor is a therapeutic strategy to treat inflammatory diseases including arthritis [45]. Neuro-inflammation of brain damage induced by permanent cerebral ischemia and renal ischemia-reperfusion injury can be protected by treatment of zileuton, a 5-LOX inhibitor [46], [47]. 5-LOX inhibitor, NDGA is reported to attenuate ovalbumin-induced lung inflammation in rats [48]. Oral administration with PF-4191834, which is developed by Pfizer, is able to ameliorate arthritis-associated pain and inflammation in rat model [11]. In addition, lecofelone, a COX/5-LOX inhibitor is reported to inhibit the progress of osteoarthritis [49]. It has also been demonstrated that PGE_2_ and LTB4 were increased in collagen induced arthritis (CIA) paws, and combination of COX-2 and 5-LOX inhibitors is able to inhibit the development of CIA [50]. In RASF, TNF-α upregulates the secretion of PGE_2_ and IL-6, and IL-6 can also be elevated by the addition of PGE_2_ [51]. Furthermore, TNF-α-induced IL-6 expression was reduced by the treatment of selective COX-2 inhibitor NS-398. However, PGE_2_ alone does not increase the expression of MCP-1 in RASF [52]. These results reveal that both 5-LOX and COX are involved in the TNF-α-induced up-regulation of cytokines. Other factors may also be related to MCP-1 expression [53], [54].

**Figure 8:**
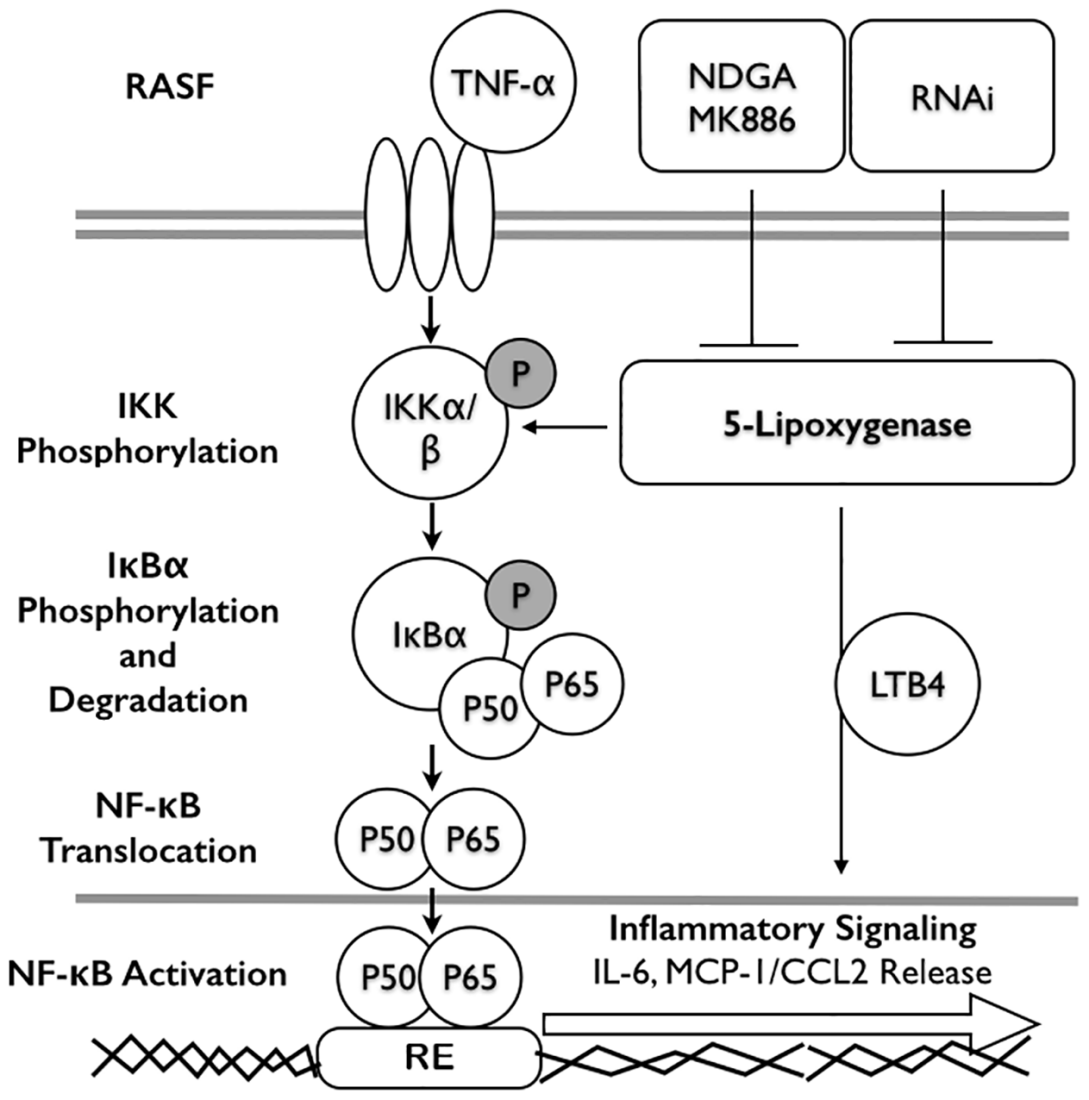
Schematic diagram of the involvement of 5-LOX in TNF-α-induced cytokine/chemokine release. TNF-α increased the release of IL-6 and MCP-1 via the activation of NF-κB signaling in RASFs. 5-LOX inhibitors, NDGA or MK886 inhibited TNF-α-induced activation of IKK and IκBα degradation. 5-LOX inhibitors also inhibited the translocation of NF-κB subunits of p50 and p65 into nucleus (response element) and then decreased TNF-α-induced IL-6 and MCP-1 release. Knockdown of 5-LOX by RNAi exerted similar inhibitory effects.

In conclusion, 5-LOX exerts a crucial role in chronic systemic inflammation. We demonstrated that both 5-LOX inhibitors antagonized TNF-α-induced IL-6 and MCP-1 expression via inhibition of NF-κB activation in human RA synovial fibroblasts (Figure 8). Moreover, 5-LOX inhibitor can reduce the TNF-α-induced systemic inflammation and paw edema *in* *vivo*. Our results reveal a novel mechanism in TNF-α-induced inflammation and suggest new therapeutic strategies targeting 5-LOX for the treatment of rheumatoid arthritis.

## Supporting Information

Figure S1 **Leukotriene B4 receptor antagonist LY29311 reverses TNF-α-induced IκBα phosphorylation and NF-κB nuclear translocation in human synovial fibroblasts.** Human synovial fibroblasts were pre-incubated with leukotriene B4 receptor antagonist LY29311 for 1 hr and then exposed to TNF-α (10 ng/ml) for another 30 min. The whole cell lysate results show that the phosphorylation of IκBα was decreased with the treatment of LY29311 in RASF. Cytosolic and nuclear extracts were separated by NE-PER kit. Note that LY29311 significantly antagonized the nuclear translocation of NF-κB subunits of p65. C23 was used as nucleus marker.
(TIF)
