# Supplementary figures and images for: 5-Lipoxygenase Inhibitors Attenuate TNF-α-Induced Inflammation in Human Synovial Fibroblasts
Source: PLoS One. 2014 Sep 17;9(9):e107890. doi: 10.1371/journal.pone.0107890 (PMC4168259; doi:10.1371/journal.pone.0107890)

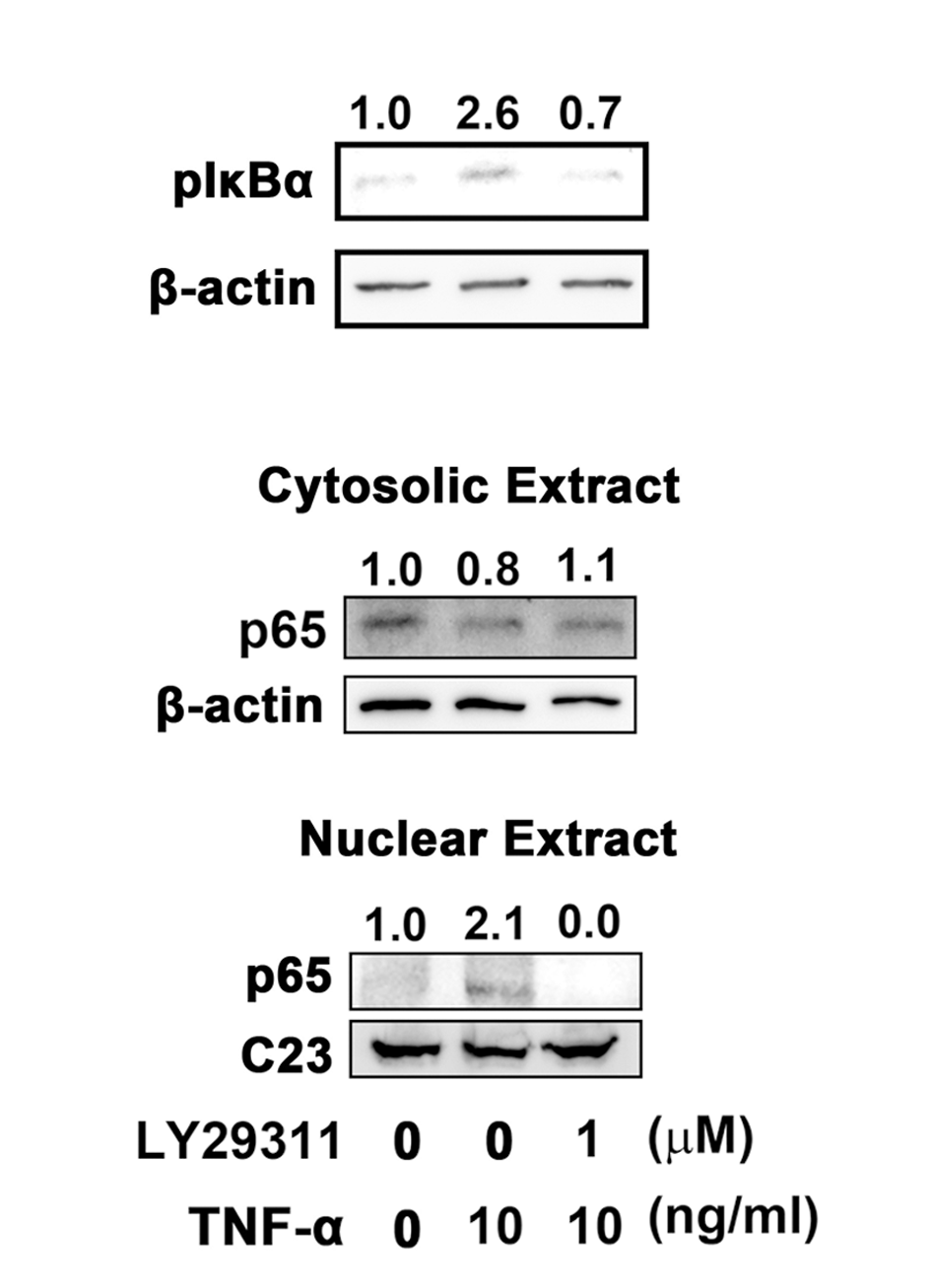

Supplement: Figure S1 — Leukotriene B4 receptor antagonist LY29311 reverses TNF-α-induced IκBα phosphorylation and NF-κB nuclear translocation in human synovial fibroblasts. Human synovial fibroblasts were pre-incubated with leukotriene B4 receptor antagonist LY29311 for 1 hr and then exposed to TNF-α (10 ng/ml) for another 30 min. The whole cell lysate results show that the phosphorylation of IκBα was decreased with the treatment of LY29311 in RASF. Cytosolic and nuclear extracts were separated by NE-PER kit. Note that LY29311 significantly antagonized the nuclear translocation of NF-κB subunits of p65. C23 was used as nucleus marker. (TIF) [file pone.0107890.s001.tif]
